# The vaccination paradox: how East Africa’s success in Ebola vaccine preparedness may be creating new public expectations for future outbreaks

**DOI:** 10.3389/fpubh.2026.1899575

**Published:** 2026-10-02

**Authors:** Okechukwu Paul-Chima Ugwu, Chinyere Nneoma Ugwu, Godson Emeka Anyanwu, Mariam Basajja, Maryann Chiamaka Ebuoh

**Affiliations:** 1 Department of Research, Publication and Extension, Kampala International University, Kampala, Uganda; 2 Department of Anatomy, Faculty of Biomedical Sciences, Kampala International University, Kampala, Uganda; 3 Department of Human Anatomy, State University of Medical and Applied Sciences, Igbo Eno, Enugu, Nigeria

**Keywords:** Bundibugyo virus disease, East Africa, Ebola vaccines, Ebola virus disease, outbreak preparedness, risk communication, Sudan virus disease, vaccine expectation gap

## Abstract

This perspective synthesizes published evidence on Ebola vaccination and outbreak preparedness in Africa. Successful vaccination against Zaire ebolavirus has strengthened vaccine-based outbreak control but may also create a preparedness risk when public expectations exceed species-specific vaccine availability. We propose the Vaccine Expectation Gap, Vaccine Anticipation and Species-Specific Vaccine Literacy as linked, hypothesis-generating constructs, supported by a Preparedness Trust Contract for practical implementation. The framework draws on expectation-disconfirmation and crisis and emergency risk communication theories and distinguishes established evidence from interpretation and testable hypotheses. Recent Sudan virus and Bundibugyo virus outbreaks illustrate the relevance of this gap because licensed vaccines remain species-specific. Evidence is concentrated in Uganda, making broader East African application a hypothesis requiring empirical testing. If validated, the framework could strengthen risk communication, community trust and regional health-security planning.

## Introduction

Ebola control has changed profoundly in the past decade. During the 2014–2016 West African Ebola epidemic, vaccines were still largely experimental. The Ebola Ça Suffit! Ring vaccination trial later demonstrated the efficacy and effectiveness of rVSV-ZEBOV against Zaire ebolavirus disease, a landmark result in modern outbreak response (1, 21). Since then, vaccination has become part of how governments, health workers, international partners and communities imagine Ebola preparedness. This success is well documented for disease caused by Zaire ebolavirus specifically. Two vaccines are approved for this indication: the single-dose rVSV-ZEBOV vaccine and the two-dose Ad26. ZEBOV/MVA-BN-Filo regimen (2, 3). These products do not automatically extend to disease caused by Sudan virus or Bundibugyo virus (3, 4).

That distinction matters in East and Central Africa, where Uganda has experienced Sudan virus disease and Bundibugyo virus disease, while the Democratic Republic of the Congo has repeatedly faced outbreaks of Zaire ebolavirus disease and, most recently, a large cross-border Bundibugyo virus disease emergency that began in May 2026 and led the World Health Organization Director-General to declare a Public Health Emergency of International Concern (4, 5). We use “Ebola virus disease” specifically for disease caused by Zaire ebolavirus, “Sudan virus disease” and “Bundibugyo virus disease” for disease caused by those respective species, and “ebolavirus disease” or “Ebola disease” as the broader umbrella term covering any recognized ebolavirus species, following current WHO usage (3).

Whether members of the public distinguish between ebolavirus species, and whether successful vaccination against one species generates expectations of availability against others, are empirical questions that, to our knowledge, have not yet been directly studied in East or Central Africa. We treat both as working hypotheses rather than established findings. If they hold, public health authorities responding to an outbreak caused by a species for which no licensed vaccine exists would face a difficult communication problem: explaining why a vaccine exists for one form of Ebola disease but not another, while maintaining trust during an active emergency. Preliminary, not-yet-peer-reviewed evidence from the 2026 Bundibugyo virus disease emergency offers some support for this concern: an analysis of public commentary on video-sharing platforms during the outbreak found that discourse was substantially shaped by institutional mistrust, misinformation and comparisons with unrelated past health emergencies, alongside expressions of trust (6). This is consistent with, but does not directly confirm, an expectation-related mistrust pathway of the kind this Perspective proposes.

This Perspective proposes that East Africa’s experience with Ebola vaccine preparedness may be creating a new public health challenge, which we term the vaccination paradox: preparedness success may increase confidence, and confidence may in turn generate expectation. Where expectations exceed what vaccine science can immediately deliver, outbreak response could face avoidable mistrust although the existence and magnitude of this effect have not yet been measured. Based on a non-systematic review of the peer-reviewed and gray literature described below, we are not aware of prior work that conceptualizes this dynamic as a distinct preparedness risk in East or Central African Ebola control; we offer this as our reading of the literature rather than as a systematic bibliometric claim. We therefore introduce a hypothesis-generating framework linking vaccine science, community expectation, risk communication and regional outbreak governance, grounded explicitly in expectation-disconfirmation theory (7) and crisis and emergency risk communication theory (8).

### Distinguishing evidence, interpretation and hypothesis

Throughout this Perspective we distinguish three categories of claim. First, established findings: facts documented in the cited primary literature, such as the existence of licensed Zaire ebolavirus vaccines or the case counts of specific outbreaks. Second, interpretations: our reading of what those findings plausibly imply, stated explicitly as interpretation. Third, hypotheses: constructs and causal pathways that this Perspective proposes but that have not been directly tested, including the Vaccine Expectation Gap, Vaccine Anticipation, and the proposed pathway from vaccine success to expectation-related mistrust. We flag hypotheses explicitly in the text, and Table 1 and Figure 1 mark the evidentiary status of each element of the framework. We recommend that reviewers, editors and readers treat these hypotheses as a research agenda rather than as demonstrated preparedness risks.

**Table 1:** The vaccine expectation gap framework for Ebola preparedness in East Africa.

Preparedness domain	Current achievement (documented)	Emerging paradox (proposed)	Strategic response (recommended)	Evidentiary status
Vaccine science	Licensed vaccines exist for Zaire ebolavirus disease	Protection may be assumed by some audiences to cover all ebolavirus disease	Promote species-specific vaccine literacy	Achievement: established finding. Paradox: proposed hypothesis
Outbreak response	Ring vaccination is a recognized Ebola control tool	Absence of a licensed Sudan virus or Bundibugyo virus vaccine may appear, to some audiences, as response failure	Communicate vaccine availability, licensing and trial status early	Achievement: established finding. Paradox: proposed hypothesis
Community confidence	Vaccine success has increased measured trust in biomedical response in surveyed populations (13)	Confidence may become unrealistic expectation	Build expectation management into preparedness communication	Achievement: established finding (specific surveyed populations). Paradox: proposed hypothesis
Research preparedness	Candidate Sudan virus vaccines have been tested during an active outbreak (16)	Trial protocols may be mistaken for routine vaccine deployment	Explain investigational use, consent and eligibility clearly	Achievement: established finding. Paradox: authors’ interpretation, partially supported by qualitative trial-perception studies (12)
Regional health security	East and Central African countries share documented outbreak and cross-border transmission risk (5)	Cross-border communities may compare unequal vaccine access	Use harmonized regional vaccine messaging	Achievement: established finding. Paradox: proposed hypothesis

The table presents a conceptual framework developed by the authors. The rightmost column, added in this revision, distinguishes claims established in the cited literature from the authors’ interpretations and from hypotheses proposed by this framework. Domains may overlap in practice and should be adapted to local outbreak histories, vaccine availability and community communication needs.

**Figure 1:**
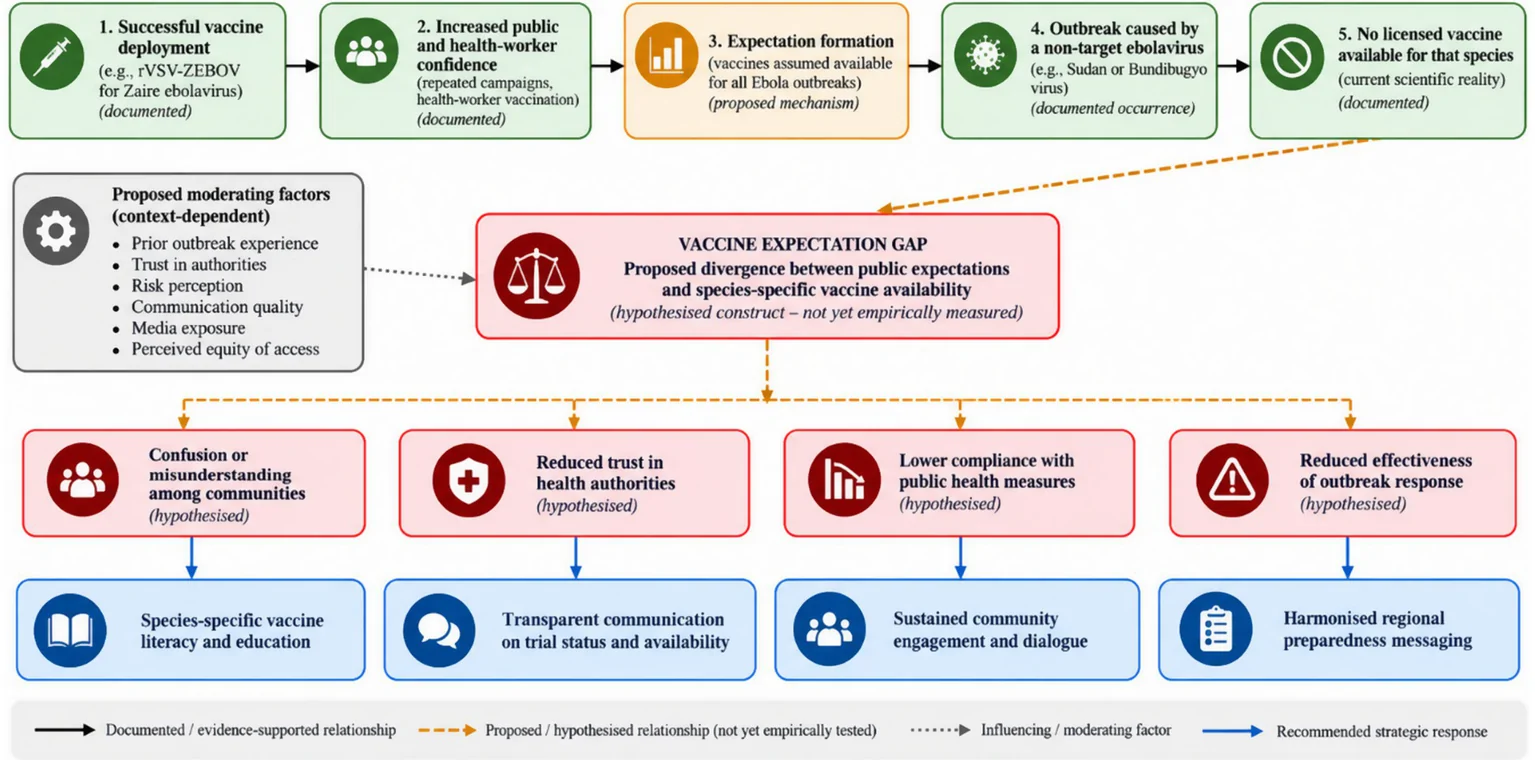
Revised conceptual framework of the Vaccine Expectation Gap in Ebola preparedness, distinguishing documented relationships from proposed, hypothesized relationships. Successful Ebola vaccine deployment against Zaire ebolavirus disease is documented to increase measured public and healthcare-worker confidence green boxes (12, 13). We hypothesize, but have not measured, that this confidence may generate an expectation that vaccines will be available for all future Ebola outbreaks (amber box). When an outbreak is caused by a non-target ebolavirus species, such as Sudan virus or Bundibugyo virus, the documented absence of a licensed vaccine may interact with this hypothesized expectation to produce a proposed Vaccine Expectation Gap (red box). We further hypothesize, without direct evidence, that this gap may produce confusion, reduced trust, lower compliance or reduced response effectiveness, moderated by factors including prior outbreak experience, communication quality, media exposure and perceived equity of access (gray box), which have not been tested in this framework. Species-specific vaccine literacy, transparent trial communication and sustained community engagement are proposed, evidence-informed strategic responses (blue boxes).

### Theoretical grounding for the proposed framework

This framework draws on four established literatures. First, expectation-disconfirmation theory proposes that satisfaction and trust are shaped by the gap between prior expectation and subsequent experience rather than by experience alone; the theory’s original empirical test used a flu-vaccination example (7), which we treat as directly analogous to, though not empirical confirmation of, the Ebola vaccine context. Second, crisis and emergency risk communication theory holds that credibility and trust during a health emergency depend on communicating uncertainty honestly and early, rather than only communicating reassurance (8); we treat species-specific vaccine literacy and transparent trial communication as applications of this principle to the Ebola vaccine context specifically. Third, the vaccine confidence literature distinguishes trust in vaccine safety and effectiveness, in the delivery system, and in policymakers’ motives (9, 10); we use this distinction below to separate Vaccine Anticipation from vaccine confidence. Fourth, empirical survey and qualitative work on Ebola vaccine perceptions in Uganda and the Democratic Republic of the Congo (11–13) documents generally high acceptance and confidence once information is provided, but also documents mistrust, rumor and perceived coercion as recurring themes; these findings motivate, without directly demonstrating, the expectation-related mistrust pathway this Perspective proposes. We are not aware of prior work that integrates these four literatures around the specific question of cross-species vaccine expectations in Ebola preparedness, which is the gap this Perspective attempts to address; again, this is our reading of a non-systematic search rather than a systematic bibliometric claim.

## Perspective scope and evidence base

This Perspective synthesizes evidence from peer-reviewed literature, World Health Organization reports, outbreak updates, vaccine policy documents, regulatory information and vaccine confidence research relevant to Ebola preparedness, with a primary empirical focus on Uganda and supporting material from the Democratic Republic of the Congo. Sources were identified through targeted, non-systematic searches of PubMed, Google Scholar, WHO publications, CDC and EMA resources, supplemented by review of outbreak-specific preprints for the ongoing 2026 Bundibugyo virus disease emergency, which had not completed peer review at the time of writing and is cited and flagged as preliminary accordingly. The objective was not to conduct a systematic review; the absence of a systematic search strategy is a limitation, and some relevant community-perception or healthcare-worker studies may not have been captured. The objective was instead to develop a conceptual, hypothesis-generating framework for understanding vaccine expectations as a candidate determinant of Ebola preparedness. Priority was given to searchable and verifiable sources, including trial reports, outbreak reports, vaccine stockpile analyses, public health guidance and recent publications on Sudan virus disease and Bundibugyo virus disease in Uganda and the Democratic Republic of the Congo.

## The vaccine expectation gap

We propose the Vaccine Expectation Gap as a hypothesized construct describing the difference between what communities may expect Ebola vaccines to provide and what available vaccines can currently deliver. We do not present this gap as demonstrated misinformation, nor as a documented phenomenon; rather, we hypothesize that it may be produced, at least in part, by successful public health messaging. When people repeatedly hear that Ebola vaccines save lives, it would not be unreasonable for them to expect vaccination during future outbreaks, although whether this expectation actually forms, and at what prevalence, has not to our knowledge been measured. The scientific reality is that Ebola vaccine protection is species-specific: a vaccine designed and licensed for Zaire ebolavirus should not be presented as a proven countermeasure for Sudan virus or Bundibugyo virus disease unless supported by evidence and regulatory approval.

We ground this construct in expectation-disconfirmation theory, which proposes that satisfaction and trust reflect the gap between prior expectation and subsequent experience rather than experience alone, and whose foundational test was itself conducted in a vaccination context (7). Applied here, the Vaccine Expectation Gap can be understood as expectation-disconfirmation theory applied to the Ebola outbreak-response encounter; whether this mechanism generalizes from consumer and routine health-service contexts to Ebola outbreak response in East and Central Africa is a hypothesis this framework proposes rather than a finding it demonstrates.

During Uganda’s Sudan virus disease outbreak in 2022, candidate vaccines were evaluated for possible inclusion in a ring vaccination trial, but no licensed Sudan virus vaccine was available for routine deployment (14). In 2025, Uganda’s Ministry of Health, WHO and partners launched the first clinical efficacy trial of a vaccine against disease caused by Sudan virus during an active outbreak (15, 16). The 2026 Bundibugyo virus disease emergency in the Democratic Republic of the Congo and Uganda, for which no licensed vaccine or approved therapeutic yet exists, provides a further, live and unfolding illustration of this proposition (4, 5), which we discuss further below. We hypothesize that the more visible Ebola vaccine preparedness becomes, the more communities may come to expect immediate vaccine deployment in any future outbreak, while the scientific and regulatory pathway remains species-specific, trial-dependent and outbreak-contingent. If this hypothesis is correct, preparedness would need to manage both vaccine stocks and public expectations; we present this as the central proposition for future empirical testing rather than as an established conclusion.

## Proposed concept 1: vaccine anticipation

Vaccine hesitancy has received substantial attention in public health literature, including work on confidence, refusal and delay (9, 10). We propose that East Africa’s Ebola context may additionally require attention to a distinct, as yet undescribed phenomenon that we term vaccine anticipation: a hypothesized state in which communities expect vaccines to be rapidly available, effective and widely deployed during an outbreak. Unlike hesitancy, anticipation, as we define it, is rooted in confidence rather than refusal; it reflects belief in biomedical preparedness. We propose that this may make it useful for outbreak response while also being fragile: if communities expect vaccination and are told that the relevant vaccine is investigational, unavailable or restricted to trial participants, confidence could, in principle, turn into disappointment. On this reading, the difficulty is not that people distrust vaccines, but that they may overestimate what currently available vaccines can cover.

### Distinguishing vaccine anticipation from related constructs

Because Vaccine Anticipation as we define it overlaps conceptually with several established constructs, Table 2 sets out our proposed distinctions. We emphasize that these constructs have not been jointly measured in the same population and that the distinctions are hypotheses for future empirical and psychometric work, not established findings.

**Table 2:** Conceptual distinctions between vaccine anticipation and related vaccine and health-response constructs.

Related construct	Core definition	Primary orientation	Proposed distinction from vaccine anticipation
Vaccine confidence	Trust in vaccine safety and effectiveness, the delivery system, and policymakers’ motives (10)	Trust-based, dispositional	Anticipation is a forward-looking belief about future availability and deployment speed, not a general trust disposition; anticipation can be high even when confidence in a specific unlicensed product is, correctly, withheld
Vaccine demand	Observed or stated desire to receive a specific, currently available vaccine	Behavioral, revealed preference	Anticipation concerns beliefs about a vaccine’s future existence or deployment for a given outbreak, independent of demand for a currently available product
Vaccine hesitancy	Delay or refusal of vaccination despite availability (9)	Refusal-oriented	Anticipation is confidence-oriented rather than refusal-oriented; we hypothesize the two occupy opposite poles of expectation rather than the same continuum
Vaccine acceptance	Willingness to receive a vaccine once it is offered (12)	Decision at point of offer	Anticipation precedes the offer; it concerns expectation before a vaccine exists or is available for a given species
Perceived vaccine availability	Belief about whether a vaccine currently exists or can be accessed	Descriptive belief	Anticipation includes an evaluative and affective component (expectation of rapid, effective deployment), not only a descriptive belief
Risk perception	Subjective assessment of personal or community vulnerability to disease	Threat appraisal	Anticipation concerns appraisal of the response (vaccine availability), not appraisal of the threat itself
Trust in health authorities	General confidence in the competence and motives of health institutions (13)	Institutional, broad	Anticipation is intervention- and outbreak-specific, whereas institutional trust is broader and more temporally stable

In this table presents the authors’ proposed conceptual distinctions. The constructs have not been jointly measured in the same population, and the distinctions are hypotheses for future testing with validated instruments, not established findings.

Preparedness programs should, in our view, ask a more precise set of questions to test these hypotheses empirically: Do communities know which Ebola vaccines exist? Do healthcare workers understand that vaccine protection differs by virus species? Do local leaders know the difference between licensed use, emergency deployment and clinical trial enrolment? We propose that these questions be addressed, and ideally measured, before the next outbreak rather than during the most anxious phase of response.

## Proposed concept 2: species-specific vaccine literacy

A second proposed dimension is species-specific vaccine literacy. Ebola communication often simplifies the disease for good reason: simple messages can save lives during outbreaks. We hypothesize, however, that oversimplification becomes risky when communities need to understand why vaccine options differ between Zaire ebolavirus, Sudan virus and Bundibugyo virus. Species-specific vaccine literacy, as we define it, does not mean teaching advanced virology; it means explaining, in locally meaningful language, that Ebola disease can be caused by related but distinct viruses, and that vaccines may work for some members of that group but not others. WHO’s current guidance makes this distinction directly: licensed vaccines and therapeutics exist for Ebola virus disease caused by Zaire ebolavirus, while approved vaccines and treatments are not available for other ebolavirus diseases such as Sudan virus disease and Bundibugyo virus disease (3). We propose that this literacy may be especially important in East and Central Africa because populations may observe different outbreak responses across borders; a community near the Uganda–DRC border may hear of vaccination in one outbreak and then wonder why vaccination is delayed or unavailable in another. We hypothesize that, without careful explanation, scientific differences of this kind risk being interpreted as neglect, secrecy or unequal access, though this interpretive pathway itself remains to be tested.

### Operationalizing species-specific vaccine literacy

We propose that species-specific vaccine literacy be assessed, in future empirical work, across five audience-specific domains, each amenable to simple knowledge or attitude items:

- Community members: recognizing that Ebola disease can be caused by more than one virus species, and that vaccine availability may differ between an outbreak they have heard about and one they are experiencing.
- Healthcare workers: recognizing which licensed vaccine, if any, corresponds to the ebolavirus species confirmed in an active outbreak, and being able to explain this distinction to patients and communities in plain language.
- Journalists and media personnel: accurately reporting the licensing and species-specific status of vaccines used or proposed in a given outbreak, avoiding generic “the Ebola vaccine” framing.
- Community and religious leaders: understanding the distinction between licensed, emergency and investigational vaccine use well enough to relay it credibly to their constituencies.
- Policymakers and outbreak coordinators: incorporating species-specific vaccine status explicitly into public communication plans and risk-communication materials before, rather than only during, an outbreak.

We propose these domains as candidate measurement targets. To our knowledge, validated instruments to assess them do not yet exist, and their development is itself a research priority arising from this framework.

## A governance mechanism: the preparedness trust contract

We present the two constructs above Vaccine Anticipation and Species-Specific Vaccine Literacy together with the Vaccine Expectation Gap, as parallel, hypothesis-generating descriptions of a single emerging phenomenon. The Preparedness Trust Contract is conceptually distinct from these three: rather than describing the phenomenon, it is our proposed governance mechanism for responding to it. We therefore treat it as a subordinate, fourth element of the framework a candidate policy and communication response to the Vaccine Expectation Gap rather than as a fourth parallel concept of the same kind as the other three. This clarifies the hierarchy of the framework: the Vaccine Expectation Gap, Vaccine Anticipation and Species-Specific Vaccine Literacy jointly describe a proposed preparedness risk; the Preparedness Trust Contract is our proposed governance response to that risk.

Vaccination also functions, we suggest, as an implicit social contract. When governments and partners invest in preparedness, communities may develop expectations about what modern outbreak response should include: rapid diagnosis, clear communication, supportive isolation, treatment, vaccination and protection for health workers. We hypothesize that when one expected component is missing, trust may weaken even if the response is scientifically appropriate, and that a Sudan virus or Bundibugyo virus disease outbreak without a licensed vaccine could therefore create a trust problem that is not primarily about vaccine refusal, but about a perceived failure to deliver protection people were led to expect. We define the Preparedness Trust Contract as the implicit agreement between public health authorities and communities that preparedness will produce timely, fair and understandable protection. In the vaccine era, we propose that this contract should explicitly include transparent communication about what vaccines exist, which virus species they target, who can receive them, whether they are licensed or investigational, and why trial protocols may be necessary.

## Uganda as the principal empirical context, with regional extension as a hypothesis

The empirical material supporting this framework is drawn principally from Uganda, supplemented by material from the Democratic Republic of the Congo. We use “East Africa” in the framing of this Perspective to describe the region in which the underlying phenomena repeated Sudan virus disease outbreaks, Bundibugyo virus disease outbreaks and cross-border Zaire ebolavirus transmission occur, and because Uganda’s experience is, to our knowledge, the most extensively documented case of a country navigating multiple ebolavirus species within a single national preparedness system. We do not claim that the Vaccine Expectation Gap has been demonstrated across East Africa as a whole. Extension of the framework to Kenya, Tanzania, Rwanda, South Sudan or other East African states, none of which has, to our knowledge, experienced a confirmed outbreak of the kind discussed here, is a hypothesis for future work rather than a documented finding. The framework describes a phenomenon documented primarily in Uganda that we hypothesize may generalize to settings combining multiple circulating ebolavirus species with an active vaccine research and preparedness program, of which the Democratic Republic of the Congo, following the 2026 Bundibugyo virus disease emergency, is a second example (5).

Uganda has experienced repeated Sudan virus disease outbreaks and a Bundibugyo virus disease outbreak, while the Democratic Republic of the Congo has experienced repeated Zaire ebolavirus disease outbreaks and, in 2026, a large Bundibugyo virus disease emergency. The 2022 Uganda outbreak resulted in 164 cases, including 142 confirmed and 22 probable cases (17, 18). In 2025, Uganda again reported Sudan virus disease, with WHO describing the absence of licensed vaccines and therapeutics for Sudan virus disease as a factor increasing public health risk (19). The region also has growing experience with Ebola vaccine logistics: the global Ebola vaccine stockpile has supported rapid access to rVSV-ZEBOV for Zaire ebolavirus outbreaks and preventive vaccination of high-risk groups (2), a major documented achievement that, we hypothesize, may also reinforce public perception that “the Ebola vaccine” is generally available. The 2025 Uganda response illustrates how the field is evolving: WHO and partners facilitated access to candidate Sudan virus vaccine doses and trial procedures at remarkable speed, with the first participants vaccinated in a WHO-led ring vaccination trial shortly after the outbreak was confirmed (15, 16). This was scientifically promising but operationally delicate, since communities may see vaccination teams and reasonably assume a proven vaccine is being deployed, while researchers and regulators understand that efficacy is still being evaluated; whether this assumption actually occurred, and at what frequency, was not measured in the sources we reviewed.

### Why Sudan virus and, increasingly, Bundibugyo virus anchor this framework

We initially anchored this framework in Uganda’s Sudan virus disease experience because it offered the most extensively documented recent case of an outbreak occurring in the absence of a licensed, species-matched vaccine, including a completed clinical efficacy trial (15, 16). Bundibugyo virus disease has a shorter documented history, with only two confirmed outbreaks prior to 2026 in Uganda in 2007–2008 and in the Democratic Republic of the Congo in 2012 which limited the case material available when earlier versions of this Perspective were drafted (20). The 2026 Bundibugyo virus disease emergency substantially expands this evidence base and is, in our assessment, now at least as important a case for this framework as the Sudan virus experience; we have incorporated it throughout this revision rather than treating Sudan virus disease as the sole anchor case.

Ebola disease caused by Bundibugyo virus was confirmed in the Democratic Republic of the Congo and Uganda in May 2026, and the WHO Director-General declared a Public Health Emergency of International Concern on 17 May 2026 (5). At the time of writing, no licensed vaccine or approved therapeutic exists specifically for Bundibugyo virus disease, although experimental evidence from non-human primate studies, human serology and monoclonal antibody research suggests that some Zaire ebolavirus-targeted vaccines and antibodies may offer partial cross-protection, supporting a “prototype pathogen” approach to preparedness even where fully species-specific products remain undeveloped (4). We read this event as a live illustration of the Vaccine Expectation Gap rather than confirmation of it: whether affected communities in fact expected a vaccine to be available, and whether its absence measurably affected trust or compliance, remain open empirical questions. Preliminary, not-yet-peer-reviewed analysis of public online discourse during the emergency found that commentary was shaped by institutional mistrust, misinformation, border-related anxiety and comparisons with COVID-19, alongside expressions of trust (6); this is consistent with, though it does not establish, the pathway this Perspective proposes.

## From vaccine hesitancy to expectation management

Most vaccine communication frameworks focus on hesitancy, misinformation and refusal. These remain important, but we propose that they are not sufficient on their own for Ebola preparedness in East and Central Africa. In settings where communities may expect vaccination, the challenge is not only to persuade people to accept vaccines; it is also to explain why a vaccine may not yet be licensed, why trial participation has eligibility criteria, and why some contacts may be offered vaccination while others are not. We propose that expectation management become a formal, testable component of Ebola vaccine preparedness, built into community engagement, healthcare-worker training and media communication before outbreaks occur, consistent with crisis and emergency risk communication principles (8). The aim, as we frame it, is not to reduce confidence in vaccines but to make confidence scientifically accurate.

## A practical framework for expectation-ready Ebola vaccination

Expectation-ready vaccination, as we conceive it, means preparing communities for both vaccine availability and vaccine gaps. We organize the following recommendations, at the suggestion of peer review, according to the outbreak preparedness cycle. We present these as recommended actions consistent with established risk-communication principles (8), rather than as interventions whose effectiveness has been tested in this specific context.

### Before an outbreak

- Develop species-specific communication materials that explain, in plain language, why vaccine availability differs between ebolavirus species.
- Train healthcare workers and media personnel on the species-specific status of licensed, investigational and candidate vaccines.
- Conduct community perception assessments to establish, empirically, whether and where a Vaccine Expectation Gap exists before assuming its presence.
- Prepare standing communication protocols for investigational-vaccine use, including plain-language explanations of consent, eligibility and randomization.

### During an outbreak

- Clearly and repeatedly distinguish licensed and investigational vaccines in all public communication.
- Explain trial eligibility and procedures in accessible language before vaccination teams are deployed.
- Provide regular, honest evidence updates, including explicit acknowledgement of what remains uncertain.
- Engage trusted community and religious leaders as communication partners rather than as one-way information recipients.

### After an outbreak

- Assess public understanding of vaccine licensing and species-specificity through structured follow-up surveys.
- Evaluate communication performance against the domains proposed in the section on species-specific vaccine literacy.
- Document lessons learned, including any evidence, positive or negative, for the Vaccine Expectation Gap hypothesis.
- Incorporate findings into national and regional preparedness plans and into future tests of this framework.

Vaccine access should, in our view, be described as both scientific and logistical: a vaccine may be unavailable because it is not licensed for the species involved, because trial approvals are required, because cold-chain systems are limited, or because the outbreak occurs in a difficult-to-reach area. We propose that East and Central Africa also consider developing regional vaccine-expectation protocols so that border communities receive harmonized messages and do not interpret differences in vaccine deployment as inequity or secrecy (Table 1).

## Discussion

We do not intend the vaccination paradox to weaken the case for Ebola vaccines; if anything, we read it as strengthening that case, since vaccines remain among the most important tools in Ebola preparedness. Our point is narrower: vaccine success may change the social environment in which outbreaks occur. Before Ebola vaccines were widely known, communities often had to be persuaded that vaccination was possible; we hypothesize that, in parts of East and Central Africa, communities may now need to understand why vaccination is not always immediately available. If this hypothesis holds, it would represent a different communication challenge requiring different preparedness tools. The Vaccine Expectation Gap, Vaccine Anticipation and Species-Specific Vaccine Literacy, together with the Preparedness Trust Contract as a subordinate governance mechanism, offer a proposed conceptual vocabulary for this problem, shifting the discussion from a narrow focus on vaccine availability toward a broader view of vaccine governance that includes science, regulation, logistics, community trust and public expectation.

As Figure 1 makes explicit, we do not claim that an expectation gap necessarily causes confusion, mistrust or reduced compliance. These outcomes plausibly depend on moderating factors such as prior outbreak experience, communication quality, media exposure, perceived equity of access and pre-existing political trust, none of which this framework has directly measured. We present the pathway from vaccine success to expectation-related mistrust as a testable hypothesis rather than a demonstrated mechanism, and we encourage future empirical work, including the community perception assessments recommended above, to test each link in this pathway rather than assuming it.

East Africa, and increasingly Central Africa, are well placed to lead this research agenda. The region has direct experience with multiple ebolavirus species, cross-border outbreak risk, healthcare-worker vulnerability and community engagement challenges, alongside growing research infrastructure and experience with vaccine trials (12). We propose that the next priority is to test, empirically, whether the constructs proposed here rather than assuming their existence can be measured and used to align vaccine science with honest, anticipatory and culturally grounded communication.

## Conclusion

Ebola vaccination has become a defining, well-documented success of modern outbreak preparedness. We propose, as a hypothesis for future testing, that this success may also create new vulnerabilities. In Uganda, and potentially more broadly in East and Central Africa, communities may increasingly expect vaccines to be part of Ebola outbreak response, while vaccine availability remains species-specific and uneven across Zaire ebolavirus, Sudan virus and Bundibugyo virus. This Perspective introduces the Vaccine Expectation Gap, Vaccine Anticipation and Species-Specific Vaccine Literacy as hypothesis-generating constructs, and the Preparedness Trust Contract as a subordinate governance mechanism, for an emerging challenge in Ebola preparedness that has not, to our knowledge, been previously conceptualized in this way. We propose that future preparedness research test whether expectations require management alongside vaccine stocks, and whether species-specific vaccine literacy and transparent communication can prevent vaccine success from unintentionally producing disappointment during outbreaks where licensed vaccines remain unavailable. We suggest that the next phase of Ebola vaccine preparedness research in East and Central Africa should test, empirically, whether combining better vaccines with deliberate expectation governance improves community trust when the next outbreak, of whichever ebolavirus species, arrives.

## Data Availability

The original contributions presented in the study are included in the article/supplementary material, further inquiries can be directed to the corresponding author.
